# Association between prescription patterns and primary care clinic closures in South Korea: A longitudinal retrospective cohort study

**DOI:** 10.1186/s12875-025-02986-9

**Published:** 2025-09-29

**Authors:** Ha Jin Kim, BeLong Cho, Jae Moon Yun

**Affiliations:** 1 Department of Family Medicine, Seoul National University Hospital, 101 Daehak-ro, Jongno-gu, Seoul, 03080 Republic of Korea; 2 Department of Family Medicine, Seoul National University College of Medicine, 101 Daehak-ro, Jongno-gu, Seoul, 03080 Republic of Korea; 3 Institute on Aging, Seoul National University College of Medicine, 71 Ihwajang-Gil, Jongno-gu, Seoul, 03087 Republic of Korea

**Keywords:** Primary health care, Prescription, Medicine

## Abstract

**Background:**

Primary care clinic sustainability affects healthcare service delivery. However, the impact of prescription patterns on primary care clinic sustainability remains unclear. We analyzed the association between prescription patterns of frequently used medications and primary care clinic closures in South Korea.

**Methods:**

We used Korean National Health Insurance Service data on office-based primary care clinics established between 2003 and 2012, and their claims data from January 1, 2002 to December 31, 2015. We assessed the effects of prescription patterns on primary care clinic closures in Korea. Prescription patterns included prescription rates of benzodiazepines and injectable medications for all visits, antibiotics for upper respiratory tract infections, and steroids for musculoskeletal diseases. We calculated these rates for the first year after the establishment of each clinic. We adjusted the association for patient-group– and clinic-related factors over 12 years. We used multivariate Cox proportional hazards regression for analyses.

**Results:**

Among 14,525 clinics, 4,681 were closed during the observation period. The average follow-up period was 5.3 years. An increase in the benzodiazepine prescription rate was associated with a lower risk of closure (hazard ratio [HR]: 0.87; 95% confidence interval [CI]: 0.82–0.92, per 10% point increase; *p* < 0.001). Steroid prescriptions for musculoskeletal diseases influenced clinic closure (HR: 0.96; 95% CI: 0.94–0.99, per 10% point increase; *p* = 0.002). Prescription rates of antibiotics for upper respiratory tract infections (HR: 1.00; 95% CI: 0.98–1.01; *p* = 0.496) and any injectable medications (HR: 0.99; 95% CI: 0.97–1.01; *p* = 0.247) did not affect primary care clinic closure.

**Conclusions:**

Steroid and benzodiazepine prescriptions were linked to lower clinic closure risk, while antibiotic and injectable prescriptions were not. These findings suggest that evidence-based prescribing may not compromise clinic viability and that appropriate care and sustainability can be achieved together through informed policy.

**Supplementary Information:**

The online version contains supplementary material available at 10.1186/s12875-025-02986-9.

## Background

Access to primary care is crucial in reducing medical expenses by ensuring the continuity and comprehensiveness of care [1]. Continuity of care is particularly essential for effective management and prognosis of chronic diseases, playing a vital role in maintaining the quality of primary care [2–4]. However, this access is influenced by the sustainability of the primary care clinics themselves. Closure of primary clinics interrupts treatment, either temporarily or consistently, severing the physician–patient relationship and disrupting the continuity of care [5]. Primary care clinic closure leads to a decline in overall health and medical accessibility for residents [6, 7], with higher rates of readmission and poorer outcomes for patients [8–10]. 

The sustainability of primary care clinics depends on various factors including financial viability, geographic accessibility, and administrative challenges. Meeting patients’ demands is often considered a potential strategy to prevent clinic closure. Among these demands, patients’ prescription preferences are believed to be particularly relevant and antibiotics for upper respiratory tract infections are a well-known example. In primary care settings, physicians may prescribe antibiotics – even when not medically indicated – in an effort to accommodate patient expectations and sustain their businesses [11, 12]. In a study conducted in Australia involving a survey of 584 general practitioners, more than half reported prescribing antibiotics for upper respiratory infections in order to meet patient demands [12]. Similar findings have been reported in studies conducted in United States and the United Kingdom [13, 14]. Reasons cited for the antibiotic prescriptions included limited consultation time, poor doctor–patient communication, diagnostic uncertainty, and concerns about clinic closure [12]. Recognizing this issue, the prescription rate in upper respiratory tract infections is one of the quality indicators suggested by the Organization for Economic Co-operation and Development (OECD) [15]. 

Similarly, other medications such as steroids for musculoskeletal diseases, benzodiazepines and injectable medications may also be prone to overprescription driven by patient expectations rather than clinical necessity. Benzodiazepines are often preferred by patients due to their rapid effects on anxiety, insomnia and managing somatic symptoms accompanied by anxiety. However, prolonged use is associated with dependence, increased risk of falls in the elderly, and challenges with discontinuation [16, 17]. Steroids are frequently prescribed for musculoskeletal pain with the expectation of quick relief, though evidence for their efficacy is limited, and their use carries risks such as joint infection, muscle atrophy, and hyperglycemia [18]. Injectable medications are also often perceived by patients as more effective than oral treatments, but inappropriate use may increase the risk of complications and healthcare costs [19, 20]. The World Health Organization suggests the injectable medication prescription rate as an appropriate prescription indicator [21]. 

Overuse of these medications may stem from the intention not to compromise the physician–patient relationship and to prevent clinic closure. However, to our knowledge, no prior studies have directly assessed whether such prescription behaviors actually influence the sustainability of primary care practices. This study aims to address that gap by evaluating the association between prescription patterns and clinic closure rates in Korea. Clarifying this relationship is essential not only for understanding clinic sustainability, but also for informing clinical and policy strategies. If meeting patient demands through potentially inappropriate prescriptions is found not to reduce closure risk, physicians may be empowered to prioritize clinical judgment over patient expectations without fear of negative business consequences. Conversely, if certain prescriptions are indeed associated with reduced closure risk, this highlights the importance of policy efforts to support appropriate prescribing and public education. In this way, our study seeks to contribute to both the sustainable delivery and quality improvement of primary care services.

## Methods

### Study population

In this longitudinal retrospective cohort study, we used data from the Korean National Health Insurance Service (NHIS) database. Since its inception in 1997, the NHIS has provided universal health coverage as a mandatory medical insurance for approximately 97% of the country’s population. The NHIS data include complete data on all medical practice performed and claimed through the National Health Insurance Corporation including patients’ sociodemographic information, medication prescriptions, and information about all medical institutions [22, 23]. 

The study population consisted of privately owned office-based primary care clinics. In Korea, the healthcare delivery system follows a three-tier structure comprising primary care clinics, general hospitals (secondary care), and tertiary general hospitals. Primary care clinics are defined as medical institutions with ≤ 30 beds or no inpatient facilities, and they can be operated by both general practitioners and specialists. As there are no specialty-based restrictions, patients have direct access to a wide range of specialties at the primary care level, e.g. ophthalmology or orthopedics, without a referral. In contrast, general hospitals provide more specialized diagnostic services and inpatient care, while tertiary general hospitals require referrals and offer advanced, highly specialized treatments.

**Fig. 1:**
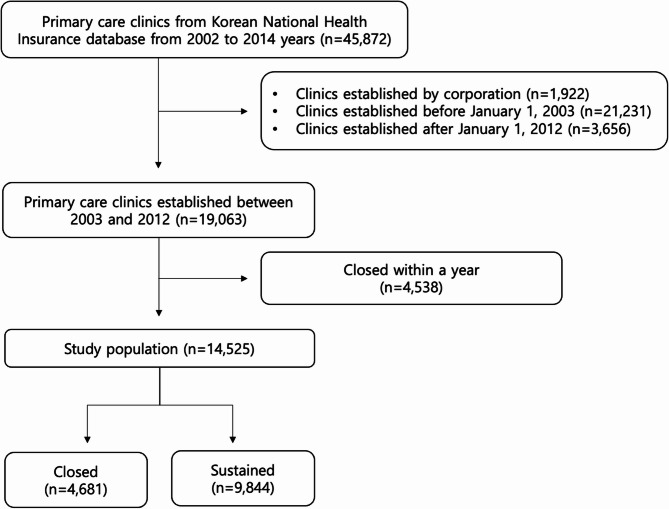
Flow chart of clinic inclusion in the study

We included newly established primary care clinics from January 1, 2003 to December 31, 2012 and followed up on clinic closure until December 31, 2014 to ensure an observation period greater than 2 years. The dataset included their claims data from January 1, 2002 to December 31, 2015. Among the initial 19,063 clinics, we excluded the clinics that closed within 1 year from their establishment date to evaluate the prescription rate for the first year after establishment. Ultimately, 14,525 primary care clinics were included in this study (Fig. 1).

### Definition of variables

#### Closure of clinics

Clinic closures were operationally defined using claims data. Primary care clinics were considered *closed* if the date of the last claim was before December 31, 2014. In contrast, the clinic was defined as *sustained* if one or more claims existed after January 1, 2015. The establishment date was defined as the date on which the first claim was made.

#### Exposures

The exposures in this study included the prescription rates of antibiotics for upper respiratory tract infections, steroids for musculoskeletal diseases, benzodiazepines, and injectable medications. We first identified patient visits for upper respiratory tract infection and musculoskeletal disease based on the International Classification of Diseases (ICD) codes J00-J06 and M13-M19, respectively. We defined the antibiotic prescription rate as the proportion of patient visits for upper respiratory tract infections that involved antibiotic prescriptions, steroid prescription rate as the proportion of patient visits that included steroid prescriptions among patient visits for musculoskeletal diseases, and prescription rates for benzodiazepine and injectable medications as the proportion of patient visits including benzodiazepine or injectable medication prescriptions among the total patient visits. The prescription rates were evaluated for 365 days after the establishment date [24]. Medication prescriptions and diagnoses were identified from the same claim corresponding to a single visit, indicating that the medications were prescribed during the same visit in which the diagnoses were recorded. We obtained prescription information from the claims data, and the diagnosis was ascertained using the ICD codes for the primary diagnosis at the visit.

#### Covariates

Risk factors related to primary care clinic closures were divided into patient group-related and clinic-related factors.

*Patient group-related factors* included age, sex, primary diagnosis for visits, insurance premium, and average medical cost of visits. Patient attributes and medical costs were evaluated based on patient visits within a year of the establishment date. The following measures were considered patient group characteristics: the proportion of patients aged ≤ 18 years, aged ≥ 65 years, and who were female. Primary diagnoses were categorized into five groups using International Classification of Diseases codes (cardiovascular [I00-I99], respiratory [J00-J99], gastrointestinal [K00-K99], endocrine [E00-E99], and musculoskeletal diseases [M00-M99]). Psychiatric diagnoses were not included in the diagnoses because the NHIS does not provide this information to protect patients’ privacy. We calculated the proportion of patient visits for these five disease categories from the total number of visits. The health insurance premium class, classified into 21 groups from 0 (Medicaid) to 20 (the highest-paying class), was included in the analysis as an indirect indicator of patients’ income and wealth. We calculated averages of overall medical costs and out-of-pocket expenses per visit. Both insurance premium class and medical costs were incorporated as continuous variables in the analysis.

*Clinic-related factors* included information about the location, professional staff, scale, and regional characteristics of the clinic. We categorized the locations into four groups: capital city, suburbs near the capital city, metropolitan cities, and others. The following factors were considered for the professional staff: representative specialties and employment of specialists (yes/no) or registered nurses (RNs) (yes/no). Scale-related factors included the number of doctors (1, 2 or more), total number of RNs and assistants (0, 1, 2, 3, 4, 5 or more), and presence of an inpatient facility (yes/no). Clinic-related information was updated annually, and the information obtained at the time of establishment was used. As proxies for regional characteristics, the number of doctors per 1,000 people in each province and the annual average insurance premium of residents were utilized as covariates. Data on the number of doctors and average insurance premiums were obtained from the NHIS for the year 2007 [25]. 

### Statistical analyses

We calculated the Clinic-Years (CYs) of observation, number of closure events, and their closure rate per 1,000 CYs according to related factors. The concept of Clinic-Year represents the sum of observation time (in years) for all included clinics, analogous to the person-year metric used in survival analyses involving individual patients. To evaluate the impact of medication prescription patterns on clinic closure rates, we performed two analyses to evaluate the effect of medication prescription patterns on the clinic closure rate. The first analysis assessed the effect of benzodiazepine and injectable medication prescription rates on clinic closure for all clinics in the study after adjustment for patient- and clinic-related factors. Upon registration of establishment, single medical specialty had to be designated as the “representative specialty” of the clinic even if physicians from multiple specialties work within the same clinic. Among the representative specialties, Pediatrics, Obstetrics, Department of Tuberculosis, Nuclear Medicine, Pathology, and the Department of Diagnostic and Testing Medicine were excluded from the analysis because they treated only specific populations or only provided diagnostic services. Subsequently, in the second analysis, antibiotics for upper respiratory tract infections and steroids for musculoskeletal diseases were added to the first analysis. This analysis was conducted on eligible clinics; clinics that did not provide care for upper respiratory tract infections or musculoskeletal diseases were excluded from the second analysis.

We used multivariate Cox proportional hazards regression for the analyses, and the most frequent group was set as a reference for each categorical variable. The hazard ratio (HR) and 95% confidence interval (CI) were calculated for every 10% point increase for continuous variables, which were calculated as percentages: proportion of patients aged ≤ 18 years, patients aged ≥ 65 years, female patients, and patients’ visit diagnoses. We calculated the HR per 1 class increase in the average health insurance premium class. The HR for insurance premiums, medical costs, and out-of-pocket costs were calculated per 100,000, 10,000, and 1,000 Korean won (approximately 0.77 USD), respectively. Less than 5% of the data were missing, and we used a complete case analysis. Statistical significance was set at *p* < 0.05. We used SAS 9.3 (SAS Institute Inc., Cary, NC, USA) for data preparation and STATA 15.0 (StataCorp LLC, TX, USA) for all analyses.

## Results

We included 14,525 clinics established between 2003 and 2012, which were observed until December 31, 2014. Table 1 displays the CYs of observation and clinic closure rate during the follow-up period. During the observation of 77,036 CYs, 4,681 clinics closed. The average follow-up period was 5.3 years. The overall primary care clinic closure rate was 60.7 per 1,000 CYs. Clinics without specialists had higher closure rates, with 105 closures per 1,000 CYs, than clinics with specialists. Of the 12,566 clinics included in the first analysis, 3,986 were closed during the follow-up period. In the second analysis, 8,322 clinics that provided care for upper respiratory tract infections and musculoskeletal diseases were included, of which 2,796 were closed during the follow-up period.

**Table 1:** Closure rates of primary care clinics according to clinic-related factors

Factor	CYs	Closure events	Rate (per 1,000 CYs)
Total	77,036	4,681	60.8
Clinic location			
Capital city	21,209	1,326	63
Suburbs of capital city	17,547	1,015	58
Metropolitan city	18,408	1,016	55
Others	19,873	1,303	66
Employment of specialists			
No	6,767	709	105
Yes	70,269	3,951	56
Employment of registered nurses			
No	54,851	3,383	62
Yes	22,186	1,277	58
Presence of inpatient facility			
No	72,177	4,288	59
Yes	4,859	372	77
Number of doctors			
1	68,492	4,070	59
2 or more	8,544	590	69
Total number of nurses			
0	4,843	309	64
1	18,601	1,315	71
2	34,267	1,867	54
3	11,389	609	53
4	3,922	253	64
5 or more	4,013	307	76

*CYs* Clinic-Years

### Prescription of antibiotics, steroids, benzodiazepines, and injectable medications

According to the analysis, higher rates of steroids for musculoskeletal diseases and benzodiazepine prescriptions were significantly associated with lower closure rates in primary care clinics after adjustment for related factors. In the second analysis, the HR for every 10% point increase in benzodiazepine prescriptions was 0.87 (95% CI: 0.82–0.92; *p* < 0.001), and the HR for every 10% point increase in steroid prescriptions was 0.96 (95% CI: 0.94–0.99; *p* = 0.002). This result was similar to that of the first analysis. Antibiotics for upper respiratory tract infections (HR: 1.00, 95% CI: 0.98–1.01; *p* = 0.496) and injectable medication prescriptions (HR: 0.99, 95% CI: 0.97–1.01; *p* = 0.247) were not associated with clinic closure (Table 2).

**Table 2:** Association between prescription rates and clinic closures

Factor	All clinics^*^		Eligible clinics^†^	
	aHR (95% CI)	*P*	aHR (95% CI)	*P*
Benzodiazepines	0.88 (0.84–0.92)	< 0.001	0.87 (0.82–0.92)	< 0.001
Any kind of injectable medication	0.99 (0.97–1.01)	0.253	0.99 (0.97–1.01)	0.247
Antibiotics for upper respiratory tract infections	N/A	N/A	1.00 (0.98–1.01)	0.496
Steroids for musculoskeletal diseases	N/A	N/A	0.96 (0.94–0.99)	0.002

*aHR* adjusted hazard ratio; *CI* confidence interval; *N/A* not applicable

^*^The first analysis assessed the effect of benzodiazepines and injectable medication prescription rates on clinic closures for all clinics (*n* = 12,566)

^†^The second analysis included prescription rates of antibiotics for upper respiratory tract infections and steroids for musculoskeletal diseases in addition to the first analysis for eligible clinics (*n* = 8,322) that provided care for upper respiratory tract infections and musculoskeletal diseases. Both analyses were adjusted for establishment year, average number of doctors per 1,000 people in the province, average insurance premium in the province, specialty, employment of specialists, employment of registered nurses, presence of inpatient facility, number of doctors, total number of registered and assistant nurses, proportion of disease, female patients, younger and older patients, income, and average of total and out-of-pocket costs

### Patient group-related factors associated with clinic closure

Clinics providing care for female patients and patients aged ≤ 18 years of age had lower risks of closure. The HRs for every 10% point increase in the proportion of female patients and patients aged ≤ 18 were 0.90 (95% CI: 0.86–0.95; *p* < 0.001) and 0.85 (95% CI: 0.81–0.90; *p* < 0.001), respectively. In contrast, an increase in the proportion of patients aged ≥ 65 years was associated with an increase in the risk of clinic closure (HR: 1.05, 95% CI: 1.01–1.10; *p* = 0.008). A higher proportion of patient visits with respiratory disease as the primary diagnosis was associated with a higher risk of clinic closure (HR: 1.05, 95% CI: 1.01–1.09; *p* = 0.008). The proportions of patient visits for other disease groups were not associated with the risk of clinic closure (Supplementary Appendix Table 1).

### Clinic-related factors associated with clinic closure

Clinics with an inpatient facility and those operated by more than one doctor had higher risks of closure than others (HR: 1.35, 95% CI: 1.16–1.56; *p* < 0.001 and HR: 1.20, 95% CI: 1.05–1.36; *p* = 0.005, respectively). Employment of specialists or RNs in clinics was not associated with clinic closure. In addition, clinics in areas with more doctors per 1,000 people had significantly lower HRs. The HR for a one doctor increase per 1,000 people in the province was 0.76. A higher average insurance premium was associated with an increased risk of closure (Supplementary Appendix Table).

## Discussion

Herein, higher prescription rates of steroids for musculoskeletal diseases and benzodiazepines were associated with the business sustainability of primary care clinics in Korea. However, clinic sustainability was less affected by antibiotic prescriptions for upper respiratory tract infections and injectable medication prescriptions. It is important to interpret these associations cautiously, as they do not imply that prescribing steroids or benzodiazepines directly prevents clinic closure. Rather, the observed relationships may reflect insufficient monitoring and limited public awareness regarding the appropriate use and potential harms of these medications.

This study findings suggest that the frequent use of antibiotics and injectable medications may no longer serve as an effective strategy for attracting or retaining patients, potentially reflecting a shift in patient preferences toward appropriate and evidence-based care rather than rapid symptomatic relief. Meanwhile, the continued association between benzodiazepine and steroid prescriptions and clinic sustainability raises concerns about the ongoing risks of overuse driven by financial or business pressures.

These results are aligned with policy efforts in Korea aimed at improving the quality of primary care. In accordance with OECD recommendations, Korea has included the rates of antibiotic and injectable medication prescriptions as key indicators in quality of care assessments [24]. As a result, the use of these medications has declined substantially over two decades [26]. However, monitoring of benzodiazepine and steroid prescriptions remains limited. Although Korea implemented a policy in 2018 restricting prescriptions of high-risk drugs, including benzodiazepines, to a maximum of 30 days, overuse and misuse of these medications remain prevalent in clinical practice [27, 28]. Furthermore, the inclusion of benzodiazepine use as a formal quality indicator is still under development, and public awareness of benzodiazepine dependence has only recently begun to gain attention [28]. The steroid prescription rate has remained constantly below 4% with quality assessments and steroid monitoring was discontinued in 2016 [26]. Accordingly, our findings underscore the need for continued monitoring of prescribing practices to prevent the detrimental effects of medication misuse and maintain the quality of primary care. Additionally, public campaigns are needed to raise awareness of appropriate medical care and promote safe prescribing behaviors. Patient-related factors showed interesting results, which can be explained by the clinics’ reputation and the variability in patient visits. Female patients or guardians of children prioritize the reputation of a clinic [29], and clinics with more female and pediatric patients and long sustainability can be presumed to have a favorable reputation. Moreover, having a high number of female and pediatric patients may indicate that the clinic is likely located near a residential area. The day-to-day variability of patient visits is expected to be lower than that of clinics in a transient area. According to our analysis by disease group, clinics with a high percentage of patients with respiratory diseases tended to have low sustainability. Respiratory diseases are seasonal epidemics that cause variability in patient visits. This variability can result in inefficient labor and operational costs, leading to problematic sustainability.

Clinic-related factors indicated that smaller scale clinics are more likely to survive in the highly competitive environment. Clinics with inpatient facilities and those operated by more than one doctor had a higher risk of closure compared to others. However, group practice is known to reduce physicians’ burnout and deliver better quality of care [30]. To address these challenges, policies that facilitate and support group practice are essential to ensure group practice sustainability and prevent physician burnout over the long term.

While this study was conducted using data from South Korea’s healthcare system, the findings may have broader relevance to other countries with similar features in their primary care environments. Korea’s primary care system allows patients to have unrestricted access to providers and operates largely under fee-for-service, creating a competitive setting for clinics. These structural characteristics may differ from systems with stronger primary care gatekeeping. However, the question of whether demand-driven prescribing supports clinic sustainability is applicable in various contexts facing pressures from patient expectations, physician autonomy in prescribing, and financial viability concerns.

Our findings suggest that clinic sustainability can be maintained without compromising evidence-based care. This conclusion may inform global efforts to promote rational prescribing while supporting practice sustainability. Future comparative studies across different healthcare systems would help further assess the generalizability of these results.

This study has several strengths. Our research was based on nationwide claims data and is the first to investigate the impact of prescription patterns on clinic closures in a longitudinal analysis. This research is particularly unique in that it includes comprehensive information about clinics and their entire medical services claimed, analyzed over more than 10 years. Previous studies were based on surveys conducted in a limited number of medical establishments with short observation periods, and without consideration of prescription patterns. Moreover, our use of claims data allowed for the inclusion of patient attributes and clinic infrastructure in the analysis, providing a more comprehensive understanding of the factors influencing clinic sustainability.

This study has limitations that should be taken into account. First, explanatory variables, including prescription behaviors and clinic characteristics, were assessed only during the first year of each clinic’s operation. Although this approach enabled a nationwide longitudinal analysis, dynamic changes over time, including change in staff, patient demographics, or prescribing patterns, could not be captured, potentially introducing temporal misclassification or residual confounding. Second, while we accounted for regional variation by categorizing clinic locations into four broad groups and included physician density per 1,000 population as a proxy for local healthcare supply, more refined measures of market competition were unavailable due to data constraints. These measures, including the number and proximity of neighboring clinics, were unavailable. Such omitted spatial factors may influence clinic sustainability through local competitive pressures. In addition, population mobility was not captured in the dataset. This may be particularly relevant in the Korean context, where there has been a long-term trend of population concentration in the capital city and its surrounding suburbs, accompanied by population decline in rural areas and some metropolitan regions. Such demographic shifts could affect clinic utilization and viability over time, especially for clinics operating in areas with declining population density. Third, individual physician characteristics (such as medical specialty, age, and clinical experience), which likely influence prescribing behavior and business sustainability, were unavailable in the dataset and thus could not be controlled for in the analysis.

Fourth, the analysis was restricted to services reimbursed by the National Health Insurance, as non-covered services, for example, elective procedures, high-cost diagnostics, cosmetic treatments, and certain injections, are not captured in claims data. These services can substantially contribute to clinic revenue and thus affect financial viability. Their exclusion may limit the comprehensiveness of our assessment. Fifth, detailed data on operational costs were not included. These costs include expenditures on rent, salaries of non-clinical staff, equipment maintenance, and depreciation. Although we incorporated certain structural indicators as indirect measures of clinic scale, the absence of direct financial variables limits the ability to assess cost-related drivers of closure.

Sixth, although we evaluated the frequency of prescriptions across specific drug categories, we were unable to determine the clinical appropriateness of these prescriptions due to the inherent limitations of claims data. In particular, the absence of diagnostic details, including the proportion of patients with psychiatric conditions, limits interpretation of psychotropic medication use. Finally, although clinic closure was used as the primary outcome, the underlying reasons for closure could not be explicitly identified. While financial difficulties are presumed to be the predominant cause, other contributing factors such as health-related issues of clinic owners or professional sanctions cannot be ruled out.

## Conclusions

This study revealed that higher prescription rates of steroids and benzodiazepines were associated with a lower risk of clinic closure, suggesting that some physicians might use these medications as a strategy to retain patients. However, antibiotic and injectable prescriptions showed no such association, indicating that evidence-based prescribing does not compromise clinic sustainability. These findings underscore the need for ongoing prescription monitoring and public education to reduce demand-driven overuse, especially for high-risk medications. Clinicians may also be reassured that avoiding unnecessary prescriptions does not threaten clinic viability, supporting safer, more appropriate care. These findings suggest that high-quality care and clinic sustainability can be achieved together through well-informed policies.

## Supplementary Information

Below is the link to the electronic supplementary material.

Supplementary Material 1

## Data Availability

The dataset utilized in this study was provided by the Korean National Health Insurance Service (NHIS), with study reference number NHIS-2017-4-025. The dataset supporting the conclusions of this article is not publicly available due to Korean government legislation. Data are available upon reasonable request and approval by the Korean National Health Insurance Service (https://nhiss.nhis.or.kr).

## Abbreviations

HR: hazard ratio
CI: confidence interval
OECD: Organization for Economic Co-operation and Development
NHIS: Korean National Health Insurance Service
ICD: International Classification of Diseases
RN: registered nurse
CYs: Clinic-Years
