# Supplementary material for: Association between prescription patterns and primary care clinic closures in South Korea: A longitudinal retrospective cohort study
Source: BMC Prim Care. 2025 Sep 29;26:293. doi: 10.1186/s12875-025-02986-9 (PMC12482020; doi:10.1186/s12875-025-02986-9)
Supplement: Supplementary file 1 — Supplementary Material 1 [file 12875_2025_2986_MOESM1_ESM.docx]

**APPENDIX I**

| **Appendix Table 1. Factors associated with clinic closures other than prescription rates** | | | | |
| --- | --- | --- | --- | --- |
| **Factors** | **All clinics^*^** | | **Eligible clinics^†^** | |
|  | **aHR (95% CI)** | ***P*** | **aHR (95% CI)** | ***P*** |
| **Patient group-related factors** | | | | |
| Proportion of female patients | 0.94 (0.91–0.96) | <0.001 | 0.90 (0.86–0.95) | <0.001 |
| Proportion of patients aged  ≤18 years | 0.86 (0.83–0.89) | <0.001 | 0.85 (0.81–0.90) | <0.001 |
| Proportion of patients aged  ≥65 years | 1.04 (1.01–1.08) | 0.009 | 1.05 (1.01–1.10) | 0.008 |
| Proportion for disease category |  |  |  |  |
| Cardiovascular disease | 0.98 (0.94–1.03) | 0.383 | 0.99 (0.93-1.06) | 0.871 |
| Endocrine disease | 0.91(0.82–1.00) | 0.056 | 0.88 (0.78-1.01) | 0.065 |
| Respiratory disease | 1.04 (1.01–1.06) | 0.005 | 1.05 (1.01-1.09) | 0.008 |
| Gastrointestinal disease | 0.96 (0.90–1.01) | 0.130 | 0.93 (0.87-1.00) | 0.044 |
| Musculoskeletal disease | 1.00 (0.98–1.02) | 0.934 | 1.01 (0.98-1.04) | 0.464 |
| Insurance premium class | 1.03 (1.00–1.06) | 0.027 | 1.02 (0.99–1.06) | 0.193 |
| Average medical cost | 1.00 (1.00–1.00) | 0.098 | 1.00 (1.00–1.00) | 0.437 |
| Average out-of-pocket cost | 0.99 (0.98–1.00) | 0.144 | 1.00 (0.98–1.01) | 0.635 |
| **Clinic-related factors** | | | | |
| Employment of specialists |  |  |  |  |
| No | 1.09 (1.00–1.20) | 0.061 | 1.03 (0.93-1.15) | 0.527 |
| Yes | (Reference) |  | (Reference) |  |
| Employment of registered  nurses |  |  |  |  |
| No | (Reference) |  | (Reference) |  |
| Yes | 0.97 (0.90–1.05) | 0.423 | 0.95 (0.87-1.04) | 0.262 |
| Presence of inpatient facility |  |  |  |  |
| No | (Reference) |  | (Reference) |  |
| Yes | 1.27 (1.11–1.46) | 0.001 | 1.35 (1.16-1.56) | <0.001 |
| Number of doctors |  |  |  |  |
| 1 | (Reference) |  | (Reference) |  |
| 2 or more | 1.19 (1.07–1.32) | 0.001 | 1.20 (1.05-1.36) | 0.005 |
| Total number of nurses |  |  |  |  |
| 0 | 0.92 (0.80–1.05) | 0.200 | 0.87 (0.73-1.04) | 0.119 |
| 1 | 1.09 (1.00–1.17) | 0.039 | 1.14 (1.04-1.25) | 0.006 |
| 2 | (Reference) |  | (Reference) |  |
| 3 | 0.97 (0.87–1.07) | 0.534 | 0.98 (0.86-1.11) | 0.721 |
| 4 | 1.12 (0.97–1.30) | 0.132 | 1.10 (0.92-1.32) | 0.309 |
| 5 or more | 1.15 (0.98–1.35) | 0.088 | 1.14 (0.94-1.38) | 0.171 |
| Average number of doctors per  1,000 people in the province | 0.78 (0.68–0.89) | 0.000 | 0.76 (0.65–0.90) | 0.017 |
| Average insurance premium in  the province | 1.05 (1.01–1.08) | 0.007 | 1.05 (1.01–1.09) | 0.001 |
| aHR, adjusted hazard ratio; CI, confidence interval; N/A, not applicable.  ^*^The first analysis assessed the effect of benzodiazepine and injectable medication prescription rates on clinic closures for all clinics (n=12,566).  ^†^The second analysis included prescription rates of antibiotics for upper respiratory tract infections and steroids for musculoskeletal diseases in addition to the first analysis for eligible clinics (n=8,322) that provided care for respiratory tract infections and musculoskeletal diseases.  Both analyses include covariates: establishment year, average number of doctors per 1,000 people in the province, average insurance premium in the province, specialty, employment of specialists, employment of registered nurses, presence of inpatient facility, number of doctors, total number of registered and assistant nurses, proportion of disease, female patients, younger and older patients, income, and average of total and out-of-pocket cost. The aHR was calculated per 10 percentage point increase of each prescription rate and variable of proportion. The HR for annual average insurance premium, average medical cost, and average out-of-pocket cost were calculated per 100,000, 10,000, and 1,000 Korean won, respectively. | | | | |

**APPENDIX II**

**Medication list used to define prescription**

1. Antibiotics

| **ATC** | **ATC_name** |
| --- | --- |
| A07AA11 | rifaximin |
| J01AA02 | doxycycline |
| J01AA06 | oxytetracycline |
| J01AA07 | tetracycline |
| J01AA08 | minocycline |
| J01AA12 | tigecycline |
| J01BA01 | chloramphenicol |
| J01BA01 | chloramphenicol |
| J01BA02 | thiamphenicol |
| J01CA01 | ampicillin |
| J01CA02 | pivampicillin |
| J01CA04 | amoxicillin |
| J01CA06 | bacampicillin |
| J01CA08 | pivmecillinam |
| J01CA12 | piperacillin |
| J01CA12 | piperacillin |
| J01CE01 | benzylpenicillin |
| J01CE08 | benzathine benzylpenicillin |
| J01CE08 | benzathine benzylpenicillin |
| J01CF06 | nafcillin |
| J01CR01 | ampicillin and enzyme inhibitor |
| J01CR02 | amoxicillin and enzyme inhibitor |
| J01CR03 | ticarcillin and enzyme inhibitor |
| J01CR04 | sultamicillin |
| J01CR05 | piperacillin and enzyme inhibitor |
| J01DB01 | cefalexin |
| J01DB03 | cefalotin |
| J01DB04 | cefazolin |
| J01DB05 | cefadroxil |
| J01DB06 | cefazedone |
| J01DB07 | cefatrizine |
| J01DB09 | cefradine |
| J01DB11 | cefroxadine |
| J01DB12 | ceftezole |
| J01DC01 | cefoxitin |
| J01DC02 | cefuroxime |
| J01DC03 | cefamandole |
| J01DC03 | cefamandole |
| J01DC04 | cefaclor |
| J01DC05 | cefotetan |
| J01DC06 | cefonicid |
| J01DC07 | cefotiam |
| J01DC08 | loracarbef |
| J01DC09 | cefmetazole |
| J01DC10 | cefprozil |
| J01DC12 | cefminox |
| J01DC13 | cefbuperazone |
| J01DC14 | flomoxef |
| J01DD | Third-generation cephalosporins |
| J01DD01 | cefotaxime |
| J01DD02 | ceftazidime |
| J01DD03 | cefsulodin |
| J01DD04 | ceftriaxone |
| J01DD04 | ceftriaxone |
| J01DD05 | cefmenoxime |
| J01DD07 | ceftizoxime |
| J01DD07 | ceftizoxime |
| J01DD08 | cefixime |
| J01DD09 | cefodizime |
| J01DD10 | cefetamet |
| J01DD11 | cefpiramide |
| J01DD12 | cefoperazone |
| J01DD13 | cefpodoxime |
| J01DD14 | ceftibuten |
| J01DD15 | cefdinir |
| J01DD16 | cefditoren |
| J01DD17 | cefcapene |
| J01DD17 | cefcapene |
| J01DD62 | cefoperazone, combinations |
| J01DE01 | cefepime |
| J01DE02 | cefpirome |
| J01DF01 | aztreonam |
| J01DF02 | carumonam |
| J01DH02 | meropenem |
| J01DH03 | ertapenem |
| J01DH04 | doripenem |
| J01DH51 | imipenem and enzyme inhibitor |
| J01DH55 | panipenem and betamipron |
| J01EC01 | sulfamethoxazole |
| J01EE01 | sulfamethoxazole and trimethoprim |
| J01EE06 | sulfadiazine and tetroxoprim |
| J01FA01 | erythromycin |
| J01FA02 | spiramycin |
| J01FA03 | midecamycin |
| J01FA06 | roxithromycin |
| J01FA07 | josamycin |
| J01FA09 | clarithromycin |
| J01FA09 | clarithromycin |
| J01FA10 | azithromycin |
| J01FA15 | telithromycin |
| J01FF01 | clindamycin |
| J01FF02 | lincomycin |
| J01GA01 | streptomycin |
| J01GB01 | tobramycin |
| J01GB04 | kanamycin |
| J01GB06 | amikacin |
| J01GB07 | netilmicin |
| J01GB08 | sisomicin |
| J01GB10 | ribostamycin |
| J01GB11 | isepamicin |
| J01GB12 | arbekacin |
| J01MA | Fluoroquinolones |
| J01MA01 | ofloxacin |
| J01MA01 | ofloxacin |
| J01MA02 | ciprofloxacin |
| J01MA03 | pefloxacin |
| J01MA06 | norfloxacin |
| J01MA06 | norfloxacin |
| J01MA07 | lomefloxacin |
| J01MA07 | lomefloxacin |
| J01MA08 | fleroxacin |
| J01MA09 | sparfloxacin |
| J01MA12 | levofloxacin |
| J01MA12 | levofloxacin |
| J01MA14 | moxifloxacin |
| J01MA15 | gemifloxacin |
| J01RA04 | spiramycin and metronidazole |
| J01XA01 | vancomycin |
| J01XA01 | vancomycin |
| J01XA02 | teicoplanin |
| J01XB01 | colistin |
| J01XC01 | fusidic acid |
| J01XC01 | fusidic acid |
| J01XC01 | fusidic acid |
| J01XD01 | metronidazole |
| J01XD01 | metronidazole |
| J01XX01 | fosfomycin |
| J01XX04 | spectinomycin |
| J01XX08 | linezolid |
| J01XX08 | linezolid |
| J04AB01 | cycloserine |
| J04AB02 | rifampicin |
| J04AB04 | rifabutin |
| J04AM05 | rifampicin, pyrazinamide and isoniazid |
| J05AB01 | aciclovir |
| NA | astromycin |
| NA | cephalexin |
| NA | cephapirin |
| NA | ciclacillin |
| NA | kitasamycin |
| NA | potassium clavulanate |
| S01AA22 | micronomicin |
| S02AA14 | gentamicin |
| S02AA15 | ciprofloxacin |

1. Benzodiazepines

| **ATC** | **ATC_name** |
| --- | --- |
| A03CA02 | clidinium and psycholeptics |
| N05AA01 | chlorpromazine |
| N05BA | Benzodiazepine derivatives |
| N05BA01 | diazepam |
| N05BA02 | chlordiazepoxide |
| N05BA06 | lorazepam |
| N05BA08 | bromazepam |
| N05BA09 | clobazam |
| N05BA12 | alprazolam |
| N05BA14 | pinazepam |
| N05BA19 | etizolam |
| N05BA21 | clotiazepam |
| N05BA23 | tofisopam |

1. Steroids

| **ATC** | **ATC_name** |
| --- | --- |
| H02AA02 | fludrocortisone |
| H02AB01 | betamethasone |
| H02AB02 | dexamethasone |
| H02AB04 | methylprednisolone |
| H02AB06 | prednisolone |
| H02AB08 | triamcinolone |
| H02AB09 | hydrocortisone |
| H02AB13 | deflazacort |
| H02BX | Corticosteroids for systemic use, combinations |

1. Injections

- Includes all the medication whose Active Ingredient Code ends with ‘BIJ’.
- Active Ingredient Code is consisted with 9 digits as below.

| Digits | ① | ② | ③ | ④ | ⑤ | ⑥ | ⑦ | ⑧ | ⑨ |
| --- | --- | --- | --- | --- | --- | --- | --- | --- | --- |
|  | Ingredient code | | | | ○ Single ingredient = dosage code  ○ Combination ingredient = 00 | | Route of Administration Code  A = Oral  B = Injection  C = Topical (external use) | Pharmaceutical  Form  Ex) tablet, capsule, injection | |

| **ATC** | **ATC_name** |
| --- | --- |
| A02BA01 | cimetidine |
| A02BA02 | ranitidine |
| A02BA03 | famotidine |
| A02BC01 | omeprazole |
| A02BC02 | pantoprazole |
| A02BC05 | esomeprazole |
| A03AB02 | glycopyrronium bromide |
| A03AC05 | tiropramide |
| A03AD01 | papaverine |
| A03AX | Other drugs for functional gastrointestinal disorders |
| A03BA01 | atropine |
| A03BB01 | butylscopolamine |
| A03BB05 | cimetropium bromide |
| A03FA01 | metoclopramide |
| A04AA | Serotonin (5HT3) antagonists |
| A04AA01 | ondansetron |
| A04AA02 | granisetron |
| A04AA05 | palonosetron |
| A04AD12 | aprepitant |
| A05BA | Liver therapy |
| A05BA06 | ornithine oxoglurate |
| A10AB01 | insulin (human) |
| A10AB04 | insulin lispro |
| A10AB05 | insulin aspart |
| A10AB06 | insulin glulisine |
| A10AC01 | insulin (human) |
| A10AD01 | insulin (human) |
| A10AD04 | insulin lispro |
| A10AD05 | insulin aspart |
| A10AD06 | insulin degludec and insulin aspart |
| A10AE04 | insulin glargine |
| A10AE05 | insulin detemir |
| A10AE06 | insulin degludec |
| A10BJ01 | exenatide |
| A10BJ03 | lixisenatide |
| A10BJ04 | albiglutide |
| A10BJ05 | dulaglutide |
| A11BA | Multivitamins, plain |
| A11CC04 | calcitriol |
| A11DA01 | thiamine (vit B1) |
| A11EA | Vitamin B-complex, plain |
| A11GA01 | ascorbic acid (vit C) |
| A11HA30 | dexpanthenol |
| A12AA03 | calcium gluconate |
| A12CC02 | magnesium sulfate |
| A16AA01 | levocarnitine |
| A16AA02 | ademetionine |
| A16AB02 | imiglucerase |
| A16AB03 | agalsidase alfa |
| A16AB04 | agalsidase beta |
| A16AB05 | laronidase |
| A16AB07 | alglucosidase alfa |
| A16AB08 | galsulfase |
| A16AB09 | idursulfase |
| A16AB10 | velaglucerase alfa |
| A16AB12 | elosulfase alfa |
| A16AX01 | thioctic acid |
| B01AB01 | heparin |
| B01AB02 | antithrombin III |
| B01AB04 | dalteparin |
| B01AB05 | enoxaparin |
| B01AB06 | nadroparin |
| B01AB12 | bemiparin |
| B01AC | Platelet aggregation inhibitors excl. heparin |
| B01AC13 | abciximab |
| B01AC17 | tirofiban |
| B01AC21 | treprostinil |
| B01AD02 | alteplase |
| B01AD04 | urokinase |
| B01AD11 | tenecteplase |
| B01AE03 | argatroban |
| B01AX | Other antithrombotic agents |
| B01AX01 | defibrotide |
| B01AX05 | fondaparinux |
| B02AA02 | tranexamic acid |
| B02AA03 | aminomethylbenzoic acid |
| B02BA01 | phytomenadione |
| B02BB01 | fibrinogen, human |
| B02BD02 | coagulation factor VIII |
| B02BD03 | factor VIII inhibitor bypassing activity |
| B02BD04 | coagulation factor IX |
| B02BD06 | von Willebrand factor and coagulation factor VIII in combination |
| B02BD08 | eptacog alfa (activated) |
| B02BX03 | batroxobin |
| B02BX04 | romiplostim |
| B03AC | Iron, parenteral preparations |
| B03BA04 | cobamamide |
| B03XA01 | erythropoietin |
| B03XA02 | darbepoetin alfa |
| B03XA03 | methoxy polyethylene glycol-epoetin beta |
| B05AA01 | albumin |
| B05AA05 | dextran |
| B05AA07 | hydroxyethylstarch |
| B05AX | Other blood products |
| B05BA01 | amino acids |
| B05BA02 | fat emulsions |
| B05BA03 | carbohydrates |
| B05BA10 | combinations |
| B05BB01 | electrolytes |
| B05BB02 | electrolytes with carbohydrates |
| B05BB04 | electrolytes in combination with other drugs |
| B05BC | Solutions producing osmotic diuresis |
| B05BC01 | mannitol |
| B05D | PERITONEAL DIALYTICS |
| B05XA01 | potassium chloride |
| B05XA02 | sodium bicarbonate |
| B05XA03 | sodium chloride |
| B05XA06 | potassium phosphate, incl. combinations with other potassium salts |
| B05XA07 | calcium chloride |
| B05XA30 | combinations of electrolytes |
| B05XB02 | alanyl glutamine |
| B05XC | Vitamins |
| C01AA05 | digoxin |
| C01BC04 | flecainide |
| C01BD01 | amiodarone |
| C01CA02 | isoprenaline |
| C01CA03 | norepinephrine |
| C01CA04 | dopamine |
| C01CA06 | phenylephrine |
| C01CA07 | dobutamine |
| C01CA24 | epinephrine |
| C01CE02 | milrinone |
| C01DA02 | glyceryl trinitrate |
| C01DA08 | isosorbide dinitrate |
| C01DX16 | nicorandil |
| C01EA01 | alprostadil |
| C01EB | Other cardiac preparations |
| C01EB10 | adenosine |
| C02DB02 | hydralazine |
| C02DD01 | nitroprusside |
| C03CA01 | furosemide |
| C04AB01 | phentolamine |
| C07AB09 | esmolol |
| C07AG01 | labetalol |
| C08CA04 | nicardipine |
| C08CA06 | nimodipine |
| C08DA01 | verapamil |
| C08DB01 | diltiazem |
| G02AB01 | methylergometrine |
| G02AD05 | sulprostone |
| G02CA01 | ritodrine |
| G02CX01 | atosiban |
| G03BA03 | testosterone |
| G03GA | Gonadotropins |
| G03GA01 | chorionic gonadotrophin |
| G03GA02 | human menopausal gonadotrophin |
| G03GA05 | follitropin alfa |
| G03GA06 | follitropin beta |
| G03GA07 | lutropin alfa |
| H01AA02 | tetracosactide |
| H01AC01 | somatropin |
| H01BA01 | vasopressin |
| H01BA02 | desmopressin |
| H01BA04 | terlipressin |
| H01BB02 | oxytocin |
| H01CB01 | somatostatin |
| H01CB02 | octreotide |
| H01CB03 | lanreotide |
| H01CB05 | pasireotide |
| H01CC01 | ganirelix |
| H01CC02 | cetrorelix |
| H02AB01 | betamethasone |
| H02AB02 | dexamethasone |
| H02AB04 | methylprednisolone |
| H02AB06 | prednisolone |
| H02AB08 | triamcinolone |
| H02AB09 | hydrocortisone |
| H04AA01 | glucagon |
| H05AA02 | teriparatide |
| H05BA01 | calcitonin (salmon synthetic) |
| H05BA04 | elcatonin |
| H05BX02 | paricalcitol |
| J01AA12 | tigecycline |
| J01BA01 | chloramphenicol |
| J01CA01 | ampicillin |
| J01CA04 | amoxicillin |
| J01CA12 | piperacillin |
| J01CE01 | benzylpenicillin |
| J01CE08 | benzathine benzylpenicillin |
| J01CF06 | nafcillin |
| J01CR01 | ampicillin and enzyme inhibitor |
| J01CR02 | amoxicillin and enzyme inhibitor |
| J01CR03 | ticarcillin and enzyme inhibitor |
| J01CR05 | piperacillin and enzyme inhibitor |
| J01DB03 | cefalotin |
| J01DB04 | cefazolin |
| J01DB06 | cefazedone |
| J01DB09 | cefradine |
| J01DB12 | ceftezole |
| J01DC01 | cefoxitin |
| J01DC02 | cefuroxime |
| J01DC03 | cefamandole |
| J01DC05 | cefotetan |
| J01DC07 | cefotiam |
| J01DC09 | cefmetazole |
| J01DC12 | cefminox |
| J01DC13 | cefbuperazone |
| J01DC14 | flomoxef |
| J01DD01 | cefotaxime |
| J01DD02 | ceftazidime |
| J01DD04 | ceftriaxone |
| J01DD05 | cefmenoxime |
| J01DD07 | ceftizoxime |
| J01DD09 | cefodizime |
| J01DD11 | cefpiramide |
| J01DD12 | cefoperazone |
| J01DD62 | cefoperazone, combinations |
| J01DE01 | cefepime |
| J01DE02 | cefpirome |
| J01DF01 | aztreonam |
| J01DH02 | meropenem |
| J01DH03 | ertapenem |
| J01DH04 | doripenem |
| J01DH51 | imipenem and enzyme inhibitor |
| J01EE01 | sulfamethoxazole and trimethoprim |
| J01FA09 | clarithromycin |
| J01FA10 | azithromycin |
| J01FF01 | clindamycin |
| J01FF02 | lincomycin |
| J01GA01 | streptomycin |
| J01GB01 | tobramycin |
| J01GB03 | gentamicin |
| J01GB04 | kanamycin |
| J01GB06 | amikacin |
| J01GB07 | netilmicin |
| J01GB10 | ribostamycin |
| J01GB11 | isepamicin |
| J01GB12 | arbekacin |
| J01MA01 | ofloxacin |
| J01MA02 | ciprofloxacin |
| J01MA12 | levofloxacin |
| J01MA14 | moxifloxacin |
| J01MA15 | gemifloxacin |
| J01XA01 | vancomycin |
| J01XA02 | teicoplanin |
| J01XB01 | colistin |
| J01XD01 | metronidazole |
| J01XD03 | ornidazole |
| J01XX04 | spectinomycin |
| J01XX08 | linezolid |
| J01XX11 | tedizolid |
| J02AA01 | amphotericin B |
| J02AC01 | fluconazole |
| J02AC02 | itraconazole |
| J02AC03 | voriconazole |
| J02AX04 | caspofungin |
| J02AX05 | micafungin |
| J02AX06 | anidulafungin |
| J05AB01 | aciclovir |
| J05AB06 | ganciclovir |
| J05AX07 | enfuvirtide |
| J06AA03 | snake venom antiserum |
| J06BA02 | immunoglobulins, normal human, for intravascular adm. |
| J06BB01 | anti-D (rh) immunoglobulin |
| J06BB02 | tetanus immunoglobulin |
| J06BB03 | varicella/zoster immunoglobulin |
| J06BB04 | hepatitis B immunoglobulin |
| J06BB09 | cytomegalovirus immunoglobulin |
| J06BB16 | palivizumab |
| L01AA01 | cyclophosphamide |
| L01AA03 | melphalan |
| L01AA06 | ifosfamide |
| L01AB01 | busulfan |
| L01AX04 | dacarbazine |
| L01BA01 | methotrexate |
| L01BA04 | pemetrexed |
| L01BB04 | cladribine |
| L01BB05 | fludarabine |
| L01BB06 | clofarabine |
| L01BC | Pyrimidine analogues |
| L01BC01 | cytarabine |
| L01BC02 | fluorouracil |
| L01BC05 | gemcitabine |
| L01BC07 | azacitidine |
| L01BC08 | decitabine |
| L01CA01 | vinblastine |
| L01CA02 | vincristine |
| L01CA04 | vinorelbine |
| L01CB01 | etoposide |
| L01CD01 | paclitaxel |
| L01CD02 | docetaxel |
| L01DB01 | doxorubicin |
| L01DB02 | daunorubicin |
| L01DB03 | epirubicin |
| L01DB06 | idarubicin |
| L01DB07 | mitoxantrone |
| L01DC01 | bleomycin |
| L01DC03 | mitomycin |
| L01XA01 | cisplatin |
| L01XA02 | carboplatin |
| L01XA03 | oxaliplatin |
| L01XC02 | rituximab |
| L01XC03 | trastuzumab |
| L01XC06 | cetuximab |
| L01XC07 | bevacizumab |
| L01XC12 | brentuximab vedotin |
| L01XC13 | pertuzumab |
| L01XC14 | trastuzumab emtansine |
| L01XC15 | obinutuzumab |
| L01XC17 | nivolumab |
| L01XC18 | pembrolizumab |
| L01XC19 | blinatumomab |
| L01XE09 | temsirolimus |
| L01XX | Other antineoplastic agents |
| L01XX02 | asparaginase |
| L01XX17 | topotecan |
| L01XX19 | irinotecan |
| L01XX27 | arsenic trioxide |
| L01XX32 | bortezomib |
| L01XX41 | eribulin |
| L01XX44 | aflibercept |
| L02AE02 | leuprorelin |
| L02AE03 | goserelin |
| L02AE04 | triptorelin |
| L02BX02 | degarelix |
| L03AA | Colony stimulating factors |
| L03AA02 | filgrastim |
| L03AA10 | lenograstim |
| L03AA13 | pegfilgrastim |
| L03AA14 | lipegfilgrastim |
| L03AB03 | interferon gamma |
| L03AB04 | interferon alfa-2a |
| L03AB05 | interferon alfa-2b |
| L03AB07 | interferon beta-1a |
| L03AB08 | interferon beta-1b |
| L03AB11 | peginterferon alfa-2a |
| L03AB13 | peginterferon beta-1a |
| L03AC01 | aldesleukin |
| L03AX | Other immunostimulants |
| L03AX03 | BCG vaccine |
| L03AX13 | glatiramer acetate |
| L03AX16 | plerixafor |
| L04AA04 | antithymocyte immunoglobulin (rabbit) |
| L04AA23 | natalizumab |
| L04AA24 | abatacept |
| L04AA25 | eculizumab |
| L04AA33 | vedolizumab |
| L04AA34 | alemtuzumab |
| L04AB01 | etanercept |
| L04AB02 | infliximab |
| L04AB04 | adalimumab |
| L04AB06 | golimumab |
| L04AC02 | basiliximab |
| L04AC05 | ustekinumab |
| L04AC07 | tocilizumab |
| L04AC10 | secukinumab |
| L04AD01 | ciclosporin |
| L04AD02 | tacrolimus |
| M01AB05 | diclofenac |
| M01AB15 | ketorolac |
| M01AC01 | piroxicam |
| M01AE01 | ibuprofen |
| M01AE03 | ketoprofen |
| M01AE17 | dexketoprofen |
| M02AA07 | piroxicam |
| M03AB01 | suxamethonium |
| M03AC02 | gallamine |
| M03AC03 | vecuronium |
| M03AC04 | atracurium |
| M03AC09 | rocuronium bromide |
| M03AC11 | cisatracurium |
| M03AX01 | botulinum toxin |
| M03BA03 | methocarbamol |
| M03BC01 | orphenadrine (citrate) |
| M03BX03 | pridinol |
| M03BX04 | tolperisone |
| M03CA01 | dantrolene |
| M05BA03 | pamidronic acid |
| M05BA06 | ibandronic acid |
| M05BA08 | zoledronic acid |
| M05BX | Other drugs affecting bone structure and mineralization |
| M05BX04 | denosumab |
| M09AX | Other drugs for disorders of the musculo-skeletal system |
| M09AX01 | hyaluronic acid |
| M09AX02 | chondrocytes, autologous |
| N01AF03 | thiopental |
| N01AH01 | fentanyl |
| N01AH02 | alfentanil |
| N01AH03 | sufentanil |
| N01AH06 | remifentanil |
| N01AX03 | ketamine |
| N01AX07 | etomidate |
| N01AX10 | propofol |
| N01BA02 | procaine |
| N01BA03 | tetracaine |
| N01BB01 | bupivacaine |
| N01BB02 | lidocaine |
| N01BB03 | mepivacaine |
| N01BB09 | ropivacaine |
| N01BB10 | levobupivacaine |
| N01BB51 | bupivacaine, combinations |
| N01BB52 | lidocaine, combinations |
| N01BB58 | articaine, combinations |
| N02AA01 | morphine |
| N02AA03 | hydromorphone |
| N02AA05 | oxycodone |
| N02AB02 | pethidine |
| N02AB03 | fentanyl |
| N02AD01 | pentazocine |
| N02AE01 | buprenorphine |
| N02AF01 | butorphanol |
| N02AF02 | nalbuphine |
| N02AX02 | tramadol |
| N02BA01 | acetylsalicylic acid |
| N02BE05 | propacetamol |
| N03AA02 | phenobarbital |
| N03AB02 | phenytoin |
| N03AB05 | fosphenytoin |
| N03AG01 | valproic acid |
| N03AX14 | levetiracetam |
| N04BB01 | amantadine |
| N05AD01 | haloperidol |
| N05AX08 | risperidone |
| N05AX12 | aripiprazole |
| N05AX13 | paliperidone |
| N05BA01 | diazepam |
| N05BA06 | lorazepam |
| N05CA01 | pentobarbital |
| N05CD08 | midazolam |
| N06BX | Other psychostimulants and nootropics |
| N06BX06 | citicoline |
| N06DX02 | Ginkgo folium |
| N07AA01 | neostigmine |
| N07AA02 | pyridostigmine |
| N07AX02 | choline alfoscerate |
| N07XX | Other nervous system drugs |
| P01CX01 | pentamidine isethionate |
| R03CA02 | ephedrine |
| R03DA05 | aminophylline |
| R05CB01 | acetylcysteine |
| R05CB02 | bromhexine |
| R05CB06 | ambroxol |
| R06AA07 | diphenylpyraline |
| R06AB04 | chlorphenamine |
| R06AB54 | chlorphenamine, combinations |
| R07 | OTHER RESPIRATORY SYSTEM PRODUCTS |
| R07AA | Lung surfactants |
| R07AB | Respiratory stimulants |
| R07AB01 | doxapram |
| S01EB02 | carbachol |
| S01EC01 | acetazolamide |
| S01JA01 | fluorescein |
| S01KA01 | hyaluronic acid |
| S01KA51 | hyaluronic acid, combinations |
| S01LA01 | verteporfin |
| S01LA04 | ranibizumab |
| S01LA05 | aflibercept |
| V03AB03 | edetates |
| V03AB04 | pralidoxime |
| V03AB06 | thiosulfate |
| V03AB14 | protamine |
| V03AB15 | naloxone |
| V03AB25 | flumazenil |
| V03AB32 | glutathione |
| V03AC01 | deferoxamine |
| V03AF01 | mesna |
| V03AF02 | dexrazoxane |
| V03AF03 | calcium folinate |
| V03AF04 | calcium levofolinate |
| V03AF06 | sodium folinate |
| V03AF07 | rasburicase |
| V03AF10 | sodium levofolinate |
| V04B | URINE TESTS |
| V04C | OTHER DIAGNOSTIC AGENTS |
| V04CD04 | corticorelin |
| V04CD05 | somatorelin |
| V04CF01 | tuberculin |
| V04CH02 | indigo carmine |
| V04CJ01 | thyrotropin |
| V04CJ02 | protirelin |
| V04CL | Tests for allergic diseases |
| V04CM01 | gonadorelin |
| V04CX | Other diagnostic agents |
| V07AB | Solvents and diluting agents, incl. irrigating solutions |
| V08AA05 | ioxitalamic acid |
| V08AB02 | iohexol |
| V08AB03 | ioxaglic acid |
| V08AB04 | iopamidol |
| V08AB05 | iopromide |
| V08AB07 | ioversol |
| V08AB09 | iodixanol |
| V08AB10 | iomeprol |
| V08AB11 | iobitridol |
| V08AD01 | ethyl esters of iodised fatty acids |
| V08CA01 | gadopentetic acid |
| V08CA02 | gadoteric acid |
| V08CA03 | gadodiamide |
| V08CA04 | gadoteridol |
| V08CA06 | gadoversetamide |
| V08CA08 | gadobenic acid |
| V08CA09 | gadobutrol |
| V08CA10 | gadoxetic acid |
| V09AA01 | technetium (99mTc) exametazime |
| V09AB | Iodine (123I) compounds |
| V09B | SKELETON |
| V09BA01 | technetium (99mTc) oxidronic acid |
| V09BA02 | technetium (99mTc) medronic acid |
| V09BA03 | technetium (99mTc) pyrophosphate |
| V09CA01 | technetium (99mTc) pentetic acid |
| V09CA02 | technetium (99mTc) succimer |
| V09CX | Other renal system diagnostic radiopharmaceuticals |
| V09CX04 | chromium (51Cr) edetate |
| V09DA04 | technetium (99mTc) mebrofenin |
| V09DB07 | technetium (99mTc) phytate |
| V09FX01 | technetium (99mTc) pertechnetate |
| V09FX02 | sodium iodide (123I) |
| V09GA01 | technetium (99mTc) sestamibi |
| V09GA02 | technetium (99mTc) tetrofosmin |
| V09GA04 | technetium (99mTc) human albumin |
| V09GX01 | thallium (201Tl) chloride |
| V09HX01 | gallium (67Ga) citrate |
| V09IB01 | indium (111In) pentetreotide |
| V09IX02 | iobenguane (131I) |
| V09IX04 | fludeoxyglucose (18F) |
| V09XA01 | iodine (131I) norcholesterol |
| V10BX01 | strontium (89Sr) chloride |
